# Supplementary material for: Pembrolizumab-Induced Simultaneous and Refractory Systemic Capillary Leak and Cytokine Release Syndromes: A Case Report
Source: Curr Oncol. 2025 Aug 18;32(8):469. doi: 10.3390/curroncol32080469 (PMC12384782; doi:10.3390/curroncol32080469)
Supplement: Supplementary file 1 [file curroncol-32-00469-s001.zip › Supplementary Table S1.pdf]

# Naranjo Adverse Drug Reaction Probability Scale

| **Question** | Yes | No | Do Not Know | Score |
|---|---|---|---|---|
| 1. Are there previous *conclusive* reports on this reaction? | +1 | 0 | 0 | |
| 2. Did the adverse event appear after the suspected drug was administered? | +2 | -1 | 0 | |
| 3. Did the adverse reaction improve when the drug was discontinued or a *specific* antagonist was administered? | +1 | 0 | 0 | |
| 4. Did the adverse event reappear when the drug was re-administered? | +2 | -1 | 0 | |
| 5. Are there alternative causes (other than the drug) that could on their own have caused the reaction? | -1 | +2 | 0 | |
| 6. Did the reaction reappear when a placebo was given? | -1 | +1 | 0 | |
| 7. Was the drug detected in blood (or other fluids) in concentrations known to be toxic? | +1 | 0 | 0 | |
| 8. Was the reaction more severe when the dose was increased or less severe when the dose was decreased? | +1 | 0 | 0 | |
| 9. Did the patient have a similar reaction to the same or similar drugs in *any* previous exposure? | +1 | 0 | 0 | |
| 10. Was the adverse event confirmed by any objective evidence? | +1 | 0 | 0 | |
| | | | **TOTAL SCORE:** | 7 |

*Modified from: Naranjo CA et al. A method for estimating the probability of adverse drug reactions. Clin Pharmacol Ther 1981; 30: 239-245.*

SCORE 5-8: Probable that the drug caused the reaction
