# Supplementary material for: Pembrolizumab-Induced Simultaneous and Refractory Systemic Capillary Leak and Cytokine Release Syndromes: A Case Report
Source: Curr Oncol. 2025 Aug 18;32(8):469. doi: 10.3390/curroncol32080469 (PMC12384782; doi:10.3390/curroncol32080469)
Supplement: Supplementary file 1 [file curroncol-32-00469-s001.zip › Supplementary Table S2.pdf]

Supplementary Table S2: Medication used and evolution of the patient’s weight

Date	Weight (kg)	Treatment initiated	Clinical status
2023-04-03		prednisone 70 mg DIE	Anasarca and atltered state of consciousness
2023-04-06	71,9		
2023-04-07	73,5	Methylprednisolone 60 mg DIE (1 mg/kg)	
2023-04-08	75,6		
2023-04-09	76,2		
2023-04-10	75,7		
2023-04-11	75,8		
2023-04-13	76,3	Mycophenolate mofetil 1500 mg x 2 doses	
2023-04-14	75,2	Immunoglobuline (IVIG) + Methylprednisolone 1000 mg id and Mycophenolate mofetil stopped	
2023-04-15	75	Methylprednisolone 1000 mg id	
2023-04-16	74,4	Methylprednisolone 1000 mg id	
2023-04-17	73,5	Methylprednisolone 60 mg id (1 mg/kg)	
2023-04-18	74,6		
2023-04-19	73,2	Levetiracetam 500 mg BID	
2023-04-23	68,8		clinicical deterioration
2023-04-24	67,6	IVIG and Methylprednisolone stopped--> Prendisone 50 mg id	
2023-04-25	67,5	IVIG	
2023-04-26	66	IVIG	improved state of consciousness
2024-04-27		IVIG	
2023-04-28	65,6	IVIG+ Prednisone 40 mg DIE	
2023-04-30	64,8	IVIG every week from now	
2023-05-02	63,5		clinical redeterioration
2023-05-09	62	Rituximab 1000 mg (1st dose)	
2023-05-10	63,2		
2023-05-15	65,4	Ruxolitinib 15 mg bid started	
2023-05-18	68,8		
2023-05-19	69,2	Plasma exchange (PLEX) attempted x 3 cycles	severe clinical redeterioration
2023-05-21	69,6		
2023-05-22	76,2		
2023-05-23	76,4		
2023-05-24	75		
2023-05-26	73,8		
2023-05-27	75,5		
2023-05-29	79,3		
2023-05-31	81,3		
2023-06-01	80,3	Rituximab 1000 mg (2nd dose)	
2023-06-02	**81,1**	From this date, the combination of IVIG every week and ruxolitinib plus diuretic and IV Albumine was the standard treatment	**Maximum weight reached**
2023-06-03	77,3		
2023-06-04	76,9		
2023-06-05	76,1		
2023-06-07	75,2		
2023-06-08	74,3		
2023-06-12	68,3		
2023-06-13	65,9		
2023-06-14	66,7		
2023-06-15	65,3		
2023-06-16	63,6		
2023-06-17	62,7		
2023-06-18	62,4		
2023-06-20	60,8		
2023-06-21	60,1		
2023-06-23	57,5		
2023-06-24	60		
2023-06-25	59,5		
2023-06-26	61,7		
2023-06-27	59,8		
2023-06-28	61,6		
2023-06-30	62,2	increased Ruxolitinib 20 mg bid (due to weight gain)	
2023-07-01	63,1		
2023-07-02	58,3		
2023-07-04	57,3		
2023-07-06	59,8	Lanreotide and midodrine was added to maintain effective circulating volume	
2023-07-07	57,9		
2023-07-09	57,4		
2023-07-11	57,4		
2023-07-12	58,6		
2023-07-13	59,6	Theophylline was tried	
2023-07-14	58,7		
2023-07-15	60,5		
2023-07-16	59,9		
2023-07-18	63,8		
2023-07-19	63,2		
2023-07-20	62,8		
2023-07-21	58,5		
2023-07-22	62,3		
2023-07-23	63,7		
2023-07-24	65,6	theophylline Stopped	Theophylline not well tolerated
2023-07-25	64,2		
2023-07-26	62		
2023-07-27	63,8	Axitinib 3 mg bid added	Leak capillary syndrome was more in control
2023-07-28	57,8		
2023-07-31	65,3		
2023-08-02	57,4		
2023-08-06	61,9		
2023-08-09	57,8		
2023-08-10	54,7		
2023-10-06	**49,9**		
2023-11-01			
2023-11-02	51,8		
2023-11-15		Enable to take ruxolitinib-axitinib-prednisone	cancer recurrence
2023-11-18			
2023-11-19	52,7		
2023-11-23	53,1		
2023-11-25	57,2		
2023-11-28	**68,8**		
2023-11-30			palliative care and death
