# Supplementary figures and images for: Pembrolizumab-Induced Simultaneous and Refractory Systemic Capillary Leak and Cytokine Release Syndromes: A Case Report
Source: Curr Oncol. 2025 Aug 18;32(8):469. doi: 10.3390/curroncol32080469 (PMC12384782; doi:10.3390/curroncol32080469)

Supplementary Figure S2: JAK/STAT pathway

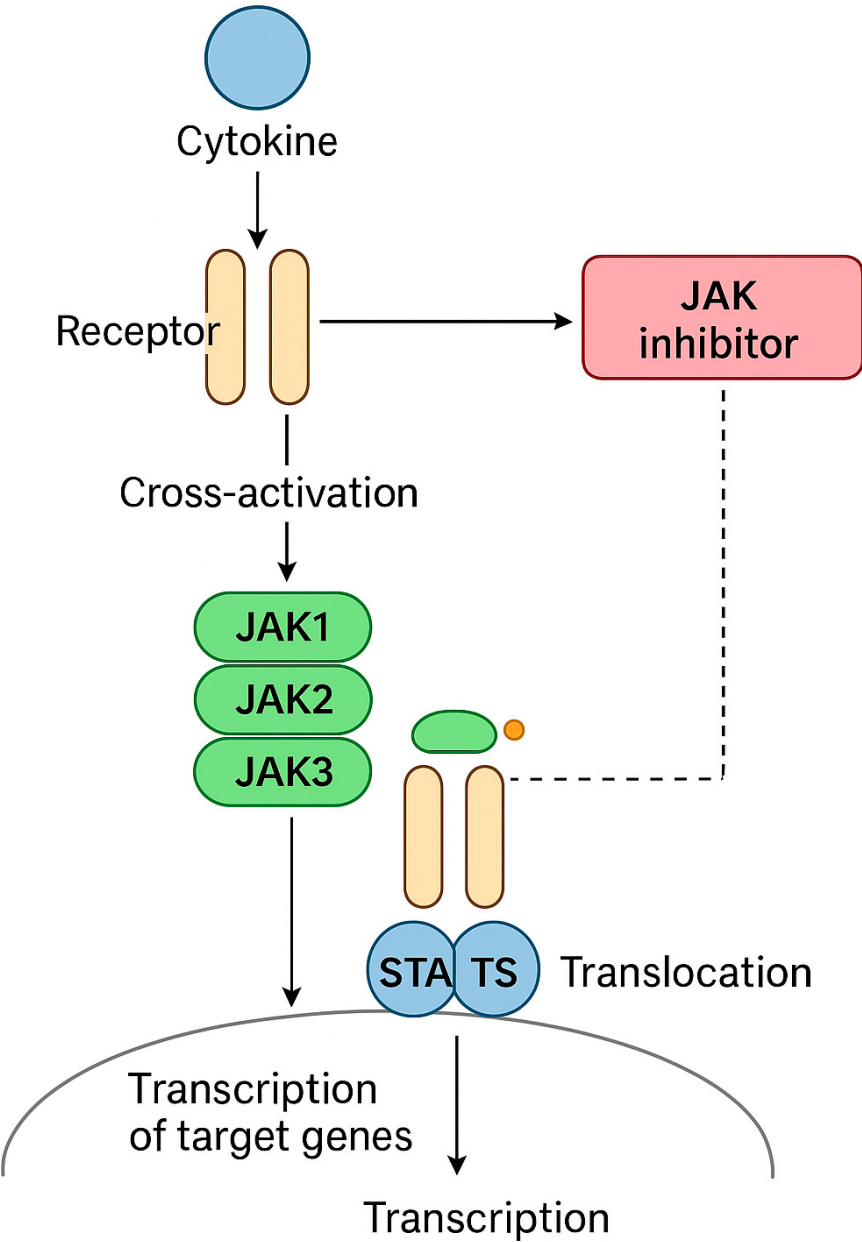

Supplement: Supplementary file 1 [file curroncol-32-00469-s001.zip › Supplementary Figure S2.pdf]
